# Lactate: an alternative pathway for the immunosuppressive properties of mesenchymal stem/stromal cells

**DOI:** 10.1186/s13287-023-03549-4

**Published:** 2023-11-19

**Authors:** Carolina Pradenas, Noymar Luque-Campos, Karina Oyarce, Rafael Contreras-Lopez, Felipe A. Bustamante-Barrientos, Andrés Bustos, Felipe Galvez-Jiron, María Jesús Araya, Catalina Asencio, Raúl Lagos, Yeimi Herrera-Luna, Daouda Abba Moussa, Charlotte Nicole Hill, Eliana Lara-Barba, Claudia Altamirano, Alexander Ortloff, Yessia Hidalgo-Fadic, Ana María Vega-Letter, María de los Ángeles García-Robles, Farida Djouad, Patricia Luz-Crawford, Roberto Elizondo-Vega

**Affiliations:** 1 Laboratorio de Biología Celular, Departamento de Biología Celular, Facultad de Ciencias Biológicas, Universidad de Concepción, Concepción, Chile; 2 Centro de Investigación Biomédica, Facultad de Medicina, Universidad de Los Andes, Santiago, Chile; 3 IMPACT, Center of Interventional Medicine for Precision and Advanced Cellular Therapy, Santiago, Chile; 4 Facultad de Medicina y Ciencia, Universidad San Sebastián, Concepción, Chile; 5 IRMB, University of Montpellier, INSERM, 34295 Montpellier, France; 6 Millennium Institute on Immunology and Immunotherapy, Santiago, Chile; 7 Escuela de Ingeniería Bioquímica, Pontificia Universidad Católica de Valparaíso, Valparaíso, Chile; 8 Departamento de Ciencias Veterinarias y Salud Pública, Facultad de Recursos Naturales, Universidad Católica de Temuco, Temuco, Chile; 9 Clinical Immunology and Osteoarticular Disease Therapeutic Unit, Department of Rheumatology, CHU Montpellier, 34095 Montpellier, France

**Keywords:** Mesenchymal stem/stromal cells, Glycolytic metabolism, Lactate dehydrogenase, lactate, Immunosuppression, Th1 and Th17 cells

## Abstract

**Background:**

The metabolic reprogramming of mesenchymal stem/stromal cells (MSC) favoring glycolysis has recently emerged as a new approach to improve their immunotherapeutic abilities. This strategy is associated with greater lactate release, and interestingly, recent studies have proposed lactate as a functional suppressive molecule, changing the old paradigm of lactate as a waste product. Therefore, we evaluated the role of lactate as an alternative mediator of MSC immunosuppressive properties and its contribution to the enhanced immunoregulatory activity of glycolytic MSCs.

**Materials and methods:**

Murine CD4^+^ T cells from C57BL/6 male mice were differentiated into proinflammatory Th1 or Th17 cells and cultured with either L-lactate, MSCs pretreated or not with the glycolytic inductor, oligomycin, and MSCs pretreated or not with a chemical inhibitor of lactate dehydrogenase A (LDHA), galloflavin or LDH siRNA to prevent lactate production. Additionally, we validated our results using human umbilical cord-derived MSCs (UC-MSCs) in a murine model of delayed type 1 hypersensitivity (DTH).

**Results:**

Our results showed that 50 mM of exogenous L-lactate inhibited the proliferation rate and phenotype of CD4^+^ T cell-derived Th1 or Th17 by 40% and 60%, respectively. Moreover, the suppressive activity of both glycolytic and basal MSCs was impaired when LDH activity was reduced. Likewise, in the DTH inflammation model, lactate production was required for MSC anti-inflammatory activity. This lactate dependent-immunosuppressive mechanism was confirmed in UC-MSCs through the inhibition of LDH, which significantly decreased their capacity to control proliferation of activated CD4^+^ and CD8^+^ human T cells by 30%.

**Conclusion:**

These findings identify a new MSC immunosuppressive pathway that is independent of the classical suppressive mechanism and demonstrated that the enhanced suppressive and therapeutic abilities of glycolytic MSCs depend at least in part on lactate production.

**Supplementary Information:**

The online version contains supplementary material available at 10.1186/s13287-023-03549-4.

## Background

Mesenchymal stem/stromal cells (MSCs) are immunosuppressive cells widely proposed as therapeutic agents for autoimmune-mediated diseases although inconsistent results between preclinical and clinical studies have been reported [1–4]. To improve the therapeutic properties of MSCs, activation with proinflammatory cytokines, such as tumor necrosis factor alpha (TNFα) and interferon-gamma (IFNγ), has been suggested [5, 6]. We have recently described that this activation significantly increases their lactate release [7]. This effect can be replicated by driving MSCs toward glycolysis using oligomycin (an inhibitor of the ATP synthase), which significantly increased MSC immunosuppressive activity on proinflammatory CD4^+^ T cells, specifically T-helper 1 and 17 (Th1 and Th17, respectively) cells while restricting inflammation in a delayed type hypersensitivity 1 (DTH) murine model [7].

Although the mechanism by which glycolytic MSCs display enhanced suppressive activity has not been elucidated, the glycolytic switch of MSCs did not alter the production of canonical suppression mediators, such as interleukin 6 (IL-6), programmed cell death ligand 1 (PD-L1), or nitric oxide (NO) [7]. However, it was associated with a significant increase in lactate release. Recently, several studies have shown that lactate acts as an immunosuppressive molecule, changing the old paradigm of lactate as a secondary metabolite waste product [8]. In mammals, the lactate produced is predominantly L-lactate in a reaction catalyzed by lactate dehydrogenase A (LDHA) [9, 10], and it can be released through members of the monocarboxylates transporters (MCT) family [11–13]. Upon its release, lactate can be taken up by other cells and used as a fuel source or display several functions, including the suppression of effector T cells [14–16]. Among the 14 known MCTs, only MCT 1–4 have been identified as lactate transporters, which are stereoselective for the transport of L-lactate [11, 17]. In addition, L-lactate can function as a signaling molecule by binding to the specific G protein-coupled hydroxycarboxylic acid receptor 1 (HCAR1) [18, 19].

The immunosuppressive effect of L-Lactate has been reported in different immune cell types including dendritic cells (DCs), natural killer (NK) cells, CD4^+^ and CD8^+^ T cells, monocytes, and macrophages [20–24]. Moreover, lactate derived from the umbilical cord-derived MSCs (UC-MSCs) promotes the differentiation and maturation of monocyte-derived DCs into M2-macrophages, suggesting that lactate might act as an immunomodulatory factor in MSCs [25]. However, no study has evaluated the role of lactate on MSC immunoregulatory properties on activated proinflammatory CD4^+^ T cells either in vitro or in vivo*.*

Therefore, in the present study, we evaluated the role of L-Lactate production by MSCs on their immunosuppressive activity using murine and human activated T cells and their potential role on the enhanced suppressive activity of glycolytic MSCs in vitro and in vivo in an inflammatory murine model of DTH.

## Methods

### Ethics statement

All experiments involving animals were conducted according to the ethical policies and procedures approved by the Ethics committee of the Faculty of Science, Universidad de Concepción, Chile (Approval CEBB-532-2019) and from the Ethics Committee of the School of Medicine at the Universidad de Los Andes in Santiago, Chile (Approval CEC 2016309). All healthy donors sign the consent forms approved by the School of Medicine at the Universidad de Los Andes in Santiago, Chile (Approval CEC 2016309). For the DTH murine model, Sixty-three adult C57BL/6 mice were supplied by the C4C animal facility. Mice were housed in a separate animal room with controlled temperature (21 ± 2 °C) and light cycle (12 h light/12 h dark). Animals were fed ad libitum with a standard rodent diet (Lab Diet, 5P00 RMH 3000, Purina Mills, St. Louis, MO) and had free access to tap water. All mice were kept within their same enriched conditions during housing. At the end of the experimental protocols, mice were euthanized by cervical dislocation under isoflurane anesthesia. Our manuscript is reported following the ARRIVE guidelines.

## MSC isolation and culture

Murine and human MSCs were isolated from either the bone marrow of C57BL/6 mice (MSC) or the human umbilical cord (UC-MSC) of newborns and characterized as previously reported [26–28]. When indicated, murine MSCs or UC-MSCs were stimulated with 1 μg/mL of oligomycin (OLN; Calbiochem, Merck, Darmstadt, Germany) for 24 h (MSC_OLN_). LDHA activity was inhibited with 50 µM galloflavin (Cayman Chemical, Ann Arbor, Michigan, USA) for 24 h (MSC_GF_ or MSC_OLN+GF_) or with 50 pmol LDHA-siRNA (Ambion Inc., Austin, Texas, USA) for 4 h (MSC_siLDHA_ or MSC_OLN+siLDHA_ or UC-MSC_siDLHA_) with the Fugene transfection reagent (Promega, Madison, Wisconsin, USA) following the manufacturer’s instructions. The validation of LDHA inhibition was performed by qPCR, lactate production measurement and by western blot analysis.

### Th1 and Th17 differentiation and immunosuppressive assay

Naïve CD4^+^ T cells were obtained using the MojoSort Mouse CD4 T Cell Isolation Kit (Biolegend, San Diego, California, USA) following the manufacturer’s instructions. Naïve CD4^+^ T cells were then stained with cell trace violet (CTV) (Life-Technology, Thermo Fisher, Carlsbad, California, USA) and cultured in a specific lymphocyte medium (MLR) in the presence of specific culture conditions to induce Th1 or Th17 cells as we previously described [28]. To test the immunosuppressive effect of lactate release by MSCs, naive CD4^+^ T cells prompted to differentiate into Th1 or Th17 were cultured alone or with 10, 25, or 50 mM L-Lactate or in the presence of MSCs under the different experimental conditions described above at a cell ratio of one MSCs per 12.5 lymphocytes in MLR medium. After 72 h, CD4^+^ T cell proliferation and differentiation were analyzed by flow cytometry.

### Flow cytometry analysis

Murine CD4^+^ T cells were stimulated for 4 h at 37 ºC with 50 ng/mL of phorbol myristate acetate (PMA) (Merck, Darmstadt, Germany), 1 mg/mL of ionomycin (Merck, Darmstadt, Germany) and 10 mg/mL of brefeldin A (Sigma-Aldrich, Merck, Darmstadt, Germany) in MLR medium as described [28]. Then, CD4^+^ T cells were stained with anti-CD25 antibodies (BD Pharmingen, San Diego, California, USA) in the presence of the LIVE/DEAD Fixable near-IR stain (Invitrogen, Thermo Fisher, Carlsbad, California, USA) and fixed with the FOXP3 Cytofix/Cytoperm buffer (eBioscience, San Diego, California, USA). Finally, the cells were labeled with intracellular fluorochrome-conjugated antibodies against IFNγ, IL17 (BD Pharmingen, USA) and FOXP3 (eBioscience, San Diego, California, USA), as previously described [7]. Flow cytometry was conducted using the BD FACS Canto II (BD Biosciences, San Diego, California, USA), and the data were analyzed with the FlowJo software (BD Biosciences, San Diego, California, USA).

### Measurement of MSC lactate production

MSCs were cultured in supplemented Dulbecco's Modified Eagle Medium (DMEM) without phenol red and fetal bovine serum (FBS) to assess inhibition of lactate production in MSCs after treatments. After 24 h, the supernatant was collected, and concentration of L-Lactate was measured with the Colorimetric Lactate Assay Kit (BioVision, Abcam, UK) according to the manufacturer’s instructions. Briefly, a standard curve was prepared with 0, 2, 4, 6, 8 and 10 nmol/well of lactate standard in a 96-well plate. Then, 1 µL of each sample and the standard curve wells were incubated for 30 min at room temperature and protected from light in a 50 µL reaction volume containing 1 µL of Lactate Enzyme Mix, 1 µL of probe, and Lactate Assay Buffer. Finally, absorbance was measured (OD, 570 nm), and lactate concentration was determined by plotting and replacing the lactate standard curve.

### Seahorse assay

The metabolism of murine MSC was determined in real time by measuring the Oxygen Consumption Rate (OCR) and the Extracellular Acidification Rate (ECAR), using the XF96 analyzer (Seahorse Biosciences, North Billerica, Massachusetts, USA) as previously described [7].

### MCT and HCAR1 expression by quantitative real-time PCR (qRT-PCR)

RNA of Th1, Th17, and naive CD4^+^ T cells were extracted using TRIzol Reagent (Invitrogen, Thermo Fisher, Carlsbad, California, USA) and then treated with DNAase I (Invitrogen, Thermo Fisher, Carlsbad, California, USA) to remove genomic DNA contamination. The purity and quantification of total RNA was measured with a NanoDrop 2000 (Thermo Scientific). Reverse transcription was performed with iScript™ cDNA Synthesis Kit (Bio-Rad, Los Angeles, California, USA) according to the manufacturer’s instructions. For qRT-PCR, 2 µL of HOT FIREPol ® EvaGreen ® qPCR Mix Plus (ROX) (Solis Biodyne, Tartu, Estonia) was used in a final volume of 10 µL containing 2 µL of cDNA diluted 1:1, 1 µM of the following sets of primers: 18S, sense 5′-GCCCGAAGCGTTTACTTTGA-3′ and antisense 5′-TTGCGCCGGTCCAAGAATTT-3′; MCT1, sense 5′-TGCAACGACCAGTGAAGTATC-3′ and antisense 5′-CAAGCCCAAGACCTCCAATAA-3′; MCT2, sense 5′- ATATTCAACACCACCTCCAGTC-3′ and antisense 5′-TGAAGCCAACGGTGAGATAAA-3′; MCT4, sense 5′- ATGAGTTTGGGATTGGCTACA-3′ and antisense 5′-GTGGTGAGGTAGATCTGGATAATG-3′; HCAR1, sense 5′-ATCCTGGTCTTCGTGCTTGG-3′ and antisense 5′- CTGTCCGAAGGGGTAAGCAG-3′. The reaction was carried out in an Mx3000P QPCR System (Agilent Technologies, USA). The relative amount of mRNA of each gene was calculated with the relative quantification method (2-ΔΔCt) and normalized according to the expression of naive CD4^+^ T cells in basal conditions.

### DTH mouse model

A DTH murine model was used to evaluate the effect of lactate inhibition in MSCs on their anti-inflammatory capacity. For that purpose, 1 mg/mL of albumin from chicken egg white (OVA) (Sigma-Aldrich, Merck, Darmstadt, Germany) in Complete Freund’s Adjuvant were intradermic injected into the lower back of C57BL/6 mice. After 5 days, paw swelling was measured and a boost of OVA in saline solution (control group) or in combination with MSCs under the different experimental conditions were injected in the hindlimb paws. After 24 h, paw thickness was measured again, and euthanasia was performed. Subpopulations of anti- or proinflammatory CD4^+^ T cells in the draining lymph nodes were determined by flow cytometry using a FACS CANTO II flow cytometer.

### Apoptosis assay

Freshly isolated naïve murine CD4^+^ T cells or human peripheral blood mononuclear cells (PBMCs) were cultured in the presence or absence of 10 mM, 20 mM or 50 mM L-Lactate for 3 days. Apoptosis was measured using Anexinn V (Invitrogen, Thermo Fisher, Carlsbad, California, USA) and propidium iodide (PI) kits (eBioscience, San Diego, California, USA), following manufacturer’s instructions, and flow cytometry using a FACS CANTO II flow cytometer.

### UC-MSCs/PBMC co-cultures

PBMCs from healthy donors (HD) were labeled with CellTrace™ Violet (Life Technologies, Thermo Fisher, Carlsbad, California, USA) and co-cultured with UC-MSCs under the different mentioned conditions in the presence of phytohemagglutinin (PHA) (5 µg/mL) (Sigma-Aldrich, Merck, Darmstadt, Germany) in MLR media. The proliferation was evaluated by FACS using a FACS CANTO II flow cytometer.

### Statistical analysis

Data are shown as the mean ± SD. All in vitro experiments were performed at least three times independently using two different biological samples each time. For the DTH murine model*,* twelve to eighteen mice per experimental group were used. The experiment was repeated three times as follows: 1st DTH experiment consisting in six animals per experimental group, including the non-treated mice induced with the DTH model, treated with murine MSCs and with murine MSCs pretreated with galloflavin. The second DTH murine model had seven animals per experimental group in the non-treated mice induced with the DTH model, treated with murine MSCs, with murine MSCs pretreated with galloflavin and murine MSCs pretreated with a siRNA against LDH. Finally, the third experiment included six animals in the non-treated DTH mice, five animals treated with murine MSCs, and eight animals treated with murine MSCs pretreated with a siRNA against LDH. Data that were identified as outlawyers were excluded. Also, 4 popliteal lymph nodes from the in vivo experiments (one non-treated DTH, one treated with murine MSC, one with siRNA against LDH and one with galloflavin) were lost during the experimental processing due to poor lymph nodes cells recovery. The p-values were generated by non-parametric analysis using the Kruskal–Wallis test for multiple comparisons and the Mann–Whitney test to compare two groups for all the data that did not fit with normal Gaussian distribution. Two-ways ANOVA and t tests were used for normal distribution. *p* < 0.05 (*), *p* < 0.01 (**) or *p* < 0.001 (***) were considered statistically significant. Data were analyzed with the GraphPad Prism TM 6 software (GraphPad Software, San Diego, California, USA).

## Results

### L-Lactate regulates the proliferation and function of Th1 and Th17 lymphocytes

**Fig. 1:**
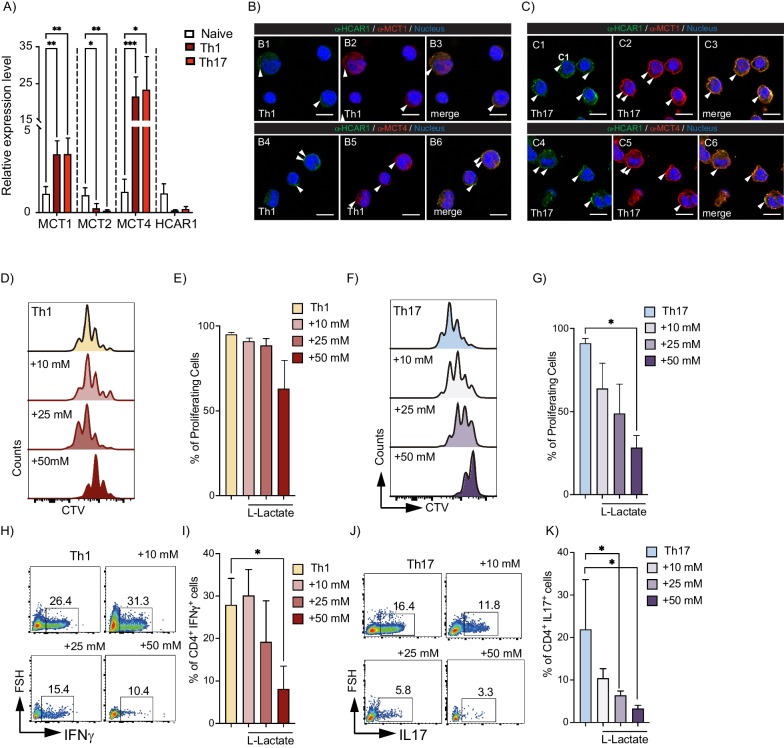
L-Lactate decreases the proliferation and differentiation of proinflammatory Th1 and Th17 cells. **A**–**C** Naïve CD4^+^ T cells from C57BL/6 mice were stained with CTV and differentiated into Th1 or Th17 cells. After 3 days of culture, relative expression of lactate receptor (HCAR1) and monocarboxylate transporters (MCTs) were analyzed by qRT-PCR (A) and immunofluorescence (**B**, **C**). Proliferation (**D**, **G**) and differentiation of naïve CD4^+^ T cells into Th1 (**H**, **I**) or Th17 (**J**, **K**) cells cultured alone or in the presence of 10, 25 or 50 mM L-Lactate were measured by flow cytometry analysis. Results represent the mean ± SD of three independent experiments with four to six biological samples; *p < 0.05, **p < 0.01, ***p < 0.001 (unpaired Kruskal–Wallis test). Unless otherwise indicated, comparisons were as compared to naïve CD4^+^ T cells for qRT-PCR and immunofluorescence or with non-treated Th1 or Th17 cells

We and others have recently described that MSC glycolytic switching significantly increases their immunosuppressive properties on murine and human derived-T cells [7, 29, 30]. Indeed, the MSCs glycolytic switch involves a substantial increase in lactate production, which could be associated with MSCs immunosuppressive capacities [7]. Therefore, to assess the role of MSCs-derived lactate on their immunomodulatory capacity, we first evaluated the expression of the lactate receptor, HCAR1, and MCTs on the different CD4^+^ T cell subsets and found a significantly higher expression level of MCT1 and MCT4 in Th1 and Th17 cells (Fig. 1A, brown and red bars, respectively) as compared with naïve CD4^+^ T cells (Fig. 1A, white bars). Immunofluorescence experiments showed that HCAR1, MCT1, and MCT4 are mainly expressed in the plasma membrane of Th17 cells and to a lesser extent in Th1 cells (Fig. 1B, C), suggesting that both Th1 and Th17 cells might be receptive to lactate stimulation. Next, we studied the proliferation and differentiation potential of naive CD4^+^ T cells into Th1 or Th17 cells in the presence of different concentrations of L-Lactate (Fig. 1D–K). Because lactate can be transported through different MCTs (e.g., MCT1 and MCT4), each with individual transport capacities given by their Km (7.7 mM and 34 mM, respectively), we chose a concentration of 10 mM L-Lactate to facilitate transport through MCT1, 25 mM to promote transport through MCT4, and 50 mM at which point, both transporters will be saturated for their transport capacity. After 3 days, 50 mM lactate induced a slight, but not significant increase in apoptosis (Additional file 1: Fig. S1A–C). At this same concentration, with no observable pH variations (data not shown), a significant decrease in the proliferation rate of both Th1 and Th17 cells was observed (Fig. 1D–G), as was the differentiation potential of CD4^+^ T cells toward Th1 (Fig. 1H, I) and Th17 cells (Fig. 1J, K). Moreover, as previously described [31], 25 mM L-Lactate significantly induced Treg cells (Additional file 1: Fig. S1D, E). Thus, these results showed that Th1 and Th17 cells express HCAR1, as well as MCT1 and MCT4 and that the exogenous addition of L-lactate significantly impairs the proliferation and differentiation process of both Th1 and Th17 cells.

### Lactate production is an essential mediator of MSC immunosuppressive activity

**Fig. 2:**
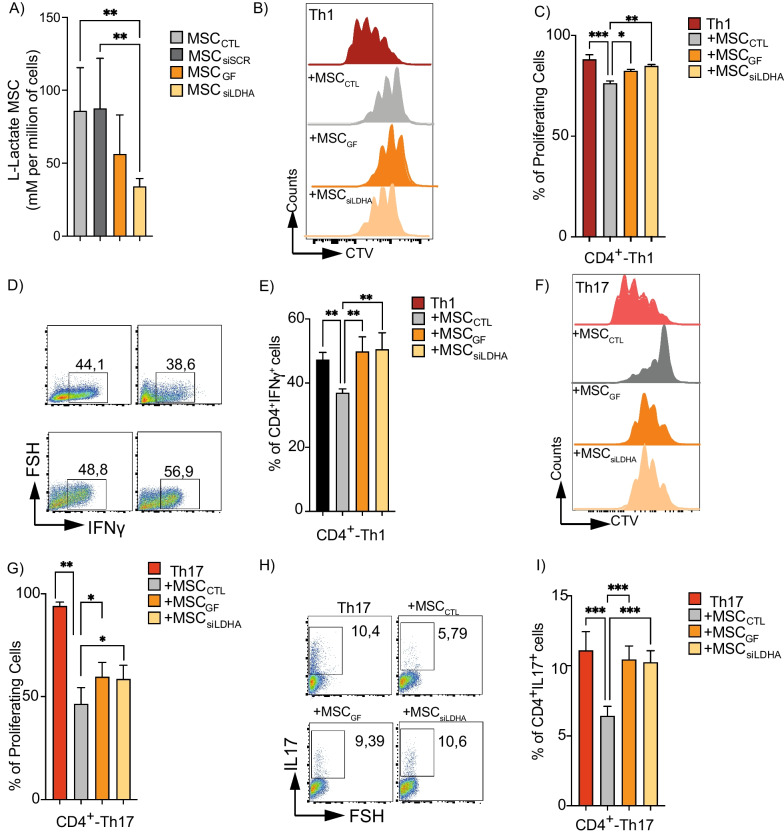
Lactate inhibition impairs the suppressive activity of murine MSCs on proinflammatory Th1 and Th17 cells. **A** Colorimetric measure of L-Lactate produced by control MSCs (gray bar), MSC_GF_ (orange bar), and MSC_siLDHA_ (yellow bar) after 24 h of treatment. **B**–**I** Naive CD4^+^ T cells from C57/BL6 mice were marked with CTV and stimulated to differentiate into Th1 (**B**–**E**) or Th17 (**F**–**I**) cells and cultured alone (light and dark red, respectively) or with control MSCs (gray bar) or pretreated with galloflavin (orange bar) or LDHA-siRNA (yellow bar) to inhibit lactate production. After 3 days of co-culture, proliferation of Th1 (**B**, **C**) and Th17 (**F**, **G**) cells, and IFNγ (**D**, **E**) or IL-17A (**H**, **I**) production were evaluated by FACS. Results represent the mean ± SD of three independent experiments lactate measurement and three independent experiments with at least six different biological samples for CD4^+^ T cells; *p < 0.05, **p < 0.01, ***p < 0.001 (unpaired Kruskal–Wallis test). Unless otherwise indicated, comparisons were with MSC in basal or control conditions, or with Th1 or Th17 cells

To determine the role of lactate produced by murine MSCs on their immunosuppressive effect, we first inhibited LDHA [32]. Using galloflavin (MSC_GF_) or a specific siRNA against LDHA (MSC_siLDHA_) (Additional file 2: Fig. S2A). After 24 h, lactate release decreased by 45% with galloflavin and 60% with siRNA, compared to naïve MSCs (MSC_CTL_) or MSCs transfected with scramble siRNA (MSC_siSCR_) (Fig. 2A). To compare the immunosuppressive capacity of these MSCs, we performed co-culture experiments with CD4^+^ T cells induced to differentiate into Th1 or Th17 cells. After 3 days of co-culture, we showed that both MSC_GF_ and MSC_siLDHA_ display a lower immunosuppressive capacity without altering the proliferation and differentiation potential of Th1 cells as compared to control MSCs (Fig. 2B–E). For Th17 cells, we observed a decrease in the suppressive effect of both MSC_GF_ and MSC_siLDHA_ on their proliferation and differentiation, as compared to MSC_CTL_ (Fig. 2F–I). No differences were observed in the generation of anti-inflammatory Treg cells for any of the experimental conditions (data not shown). This effect was not associated with a detectable metabolic switch in MSCs, since neither galloflavin nor the siRNA significantly modified the basal oxygen consumption rate (OCR) or the basal extracellular acidification rate (ECAR) (Additional file 2: Fig. S2B, C). Similarly, when we quantified the expression of the canonical molecules involved in the immunosuppressive properties of MSCs (e.g., VCAM, ICAM, PD-L1 or NO_2_, activated or not with IFNγ or TNFα), we only observed changes in the ICAM expression levels in response to LDHA inhibition under basal conditions (Additional file 3: Fig. S3A–M). To validate these results, we used a general pharmacological inhibition of MCT on MSCs using the specific inhibitors of MCT activity, AZD3965 (25 nM) and alpha-cyano-4-hydroxycinnamate (4-CIN, 1 mM) in vitro. Inhibition of lactate transporters in MSCs also impairs the suppressive activity of MSCs on the proliferation and phenotype of both Th1 (Additional file 4: Fig. S4B, C) and Th17 cells (Additional file 4: Fig. S4D, E), thus supporting the role of lactate as an MSC immunosuppressive mechanism. These results reveal that the immunomodulatory effects of MSCs on Th1 and Th17 cells are partially dependent on lactate production.

### Inhibition of lactate production impairs the immunoregulatory properties of MSC in vivo

**Fig. 3:**
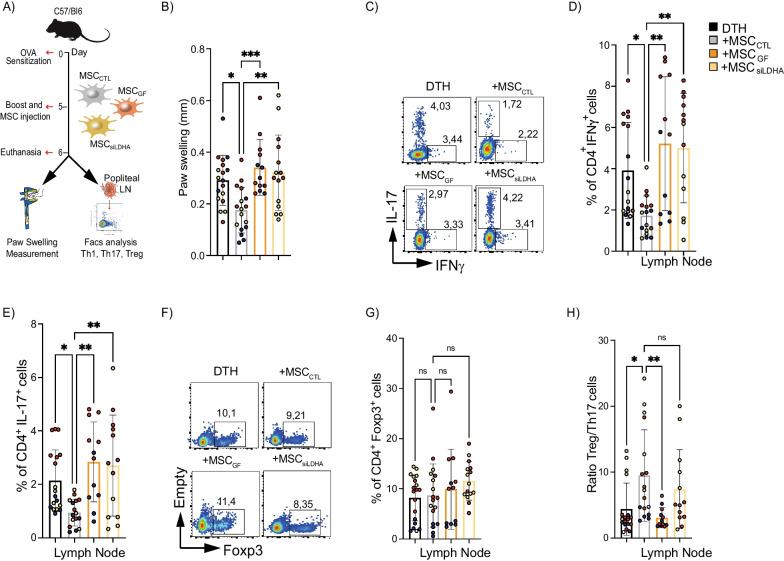
Inhibition of lactate production in MSCs reduces their immunotherapeutic activity in a murine DTH model. **A** Experimental design of the DTH murine model. **B** After 24 h post-boost and MSCs injection, paw swelling was measured on the hindlimb paws. **C**–**H** After euthanasia, proinflammatory Th1 (**C**, **D**) and Th17 (**E**, **F**) lymphocytes and anti-inflammatory Treg cells (**G**, **H**) present in the draining lymph nodes of DTH mice (black line bars) or DTH mice treated with MSC_CTL_ (gray line bars), MSC_GF_ (orange line bars) or MSC_siLDHA_ (yellow line bars) were measured by FACS. **G** Treg/Th17 ratio was also calculated. Each color represents an independent DTH experiment (DTH1: in dark blue; DTH2: in red; DTH3 in yellow). Results represent the mean ± SD of three independent experiments with at least 12 animals per experimental group; *p < 0.05, **p < 0.01, ***p < 0.001, ****p < 0.0001 (Unpaired Anova test). Unless otherwise indicated, comparisons were with untreated DTH mice

To establish the role of lactate produced by MSCs on their immunoregulatory potential in vivo, we relied on the DTH murine model (Fig. 3A), which has been extensively reported as a proinflammatory murine model, to study cell-mediated host immune response associated with T-CD4^+^ cells as measured by the localized swelling of the immunized joint [33, 34]. In the present work, we aimed to determine the effect of inhibiting lactate release in MSCs on local inflammation in the DTH model by evaluating localized swelling of the joint where the DTH was induced (paws) and the immune response in the popliteal lymph node, nearby the side of injection (Fig. 3A). Compared to untreated DTH mice, those treated with MSC_CTL_ showed a 50% decrease in the paw swelling (Fig. 3B, black and gray line bars, respectively). In contrast, mice treated with MSC_GF_ or MSC_siLDHA_ did not show any decrease in paw swelling (Fig. 3B, orange and yellow line bars, respectively). In addition, the frequency of Th1 and Th17 cells was significantly lower only in the mice treated with MSC_CTL_ as compared to the untreated DTH mice (Fig. 3C–E, gray and black line bar). This MSC-mediated immunoregulatory effect was completely lost when the lactate production was inhibited (Fig. 3C–E, orange, and yellow line bars). Of note, while no difference in the percentage of Treg cells was observed in the popliteal lymph nodes of mice in the different groups (Fig. 3F, G), a 50% increase in the Treg/Th17 ratio was observed only in the group treated with MSC_CTL_ as compared to non-treated DTH mice (Fig. 3H, gray and black line bar, respectively). To evaluate the peripheral immune response, we quantified the generation of Th1, Th17, and Treg cells in peripheral blood, which showed a significant inhibition of Th1 (Additional file 5: Fig. S5A) and Th17 cells (Additional file 5: Fig. S5B) response when mice were treated with MSCs. This effect was abrogated when MSCs were had genetic or pharmacological inhibition of LDHA. No effect was observed on Treg cells (Additional file 5: Fig. S5C). Therefore, these data showed that lactate released by MSCs is critical for their immunoregulatory potential in a murine inflammatory model.

### The enhanced immunosuppressive capacity of glycolytic MSCs depends on their lactate production

**Fig. 4:**
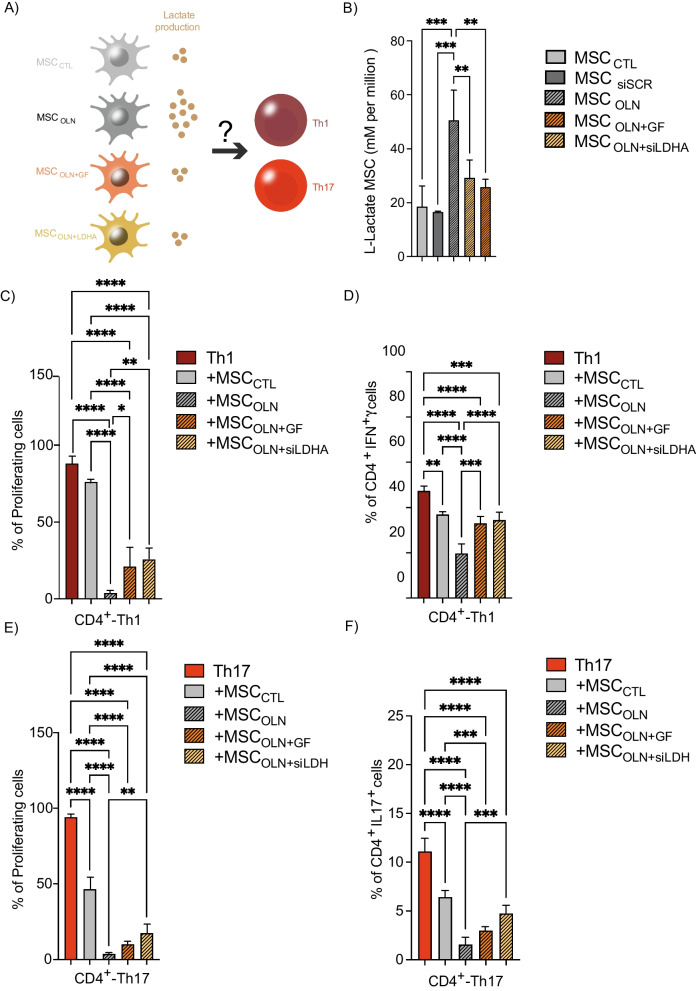
Lactate produced by glycolytic MSCs mediates their enhanced immunosuppressive effect. **A** Experimental design using MSCs pretreated or not with oligomycin alone (MSC_OLN_) or together with galloflavin (MSC_OLN+GF_) or siRNA against LDHA (MSC_OLN+siLDHA_). **B** Colorimetric analysis of MSC lactate efflux after 24 h of treatment. **C**–**F** Naive CD4^+^ T cells isolated from spleen of C57BL/6 mice were labeled with CTV and induced to differentiation into Th1 (**C**, **D** or Th17 (**E**, **F**) cells and were cultured alone (dark and light red bars, respectively) or together with MSC_CTL_ (gray bars), MSC_OLN_ (striped, gray bars), MSC_OLN+GF_ (striped orange bars), or MSC_OLN+siLDHA_ (striped yellow bars). After three days of co-culture, proliferation (**C**, **E**) and phenotype (**D**, **F**) of lymphocytes were characterized by flow cytometry. Results represent the mean ± SD of three independent experiments for lactate measurement in MSCs and three independent experiments with at least four different biological samples for CD4^+^ T cells; *p < 0.05, **p < 0.01, ***p < 0.001, ****p < 0.0001 (unpaired Kruskal–Wallis test). Unless otherwise indicated, comparisons were with MSCs in basal or control conditions or with Th1 or Th17 cells

We have previously demonstrated that the induction of the MSCs glycolytic switch using the ATP synthase inhibitor OLN significantly enhanced their immunosuppressive activity on Th1 and Th17 lymphocytes as compared to MSC_CTL_ [7]. This effect was associated with greater lactate efflux [7]. To assess whether the lactate produced by glycolytic MSCs is associated with their enhanced immunomodulatory capacity, we either pretreated murine MSCs with OLN alone (MSC_OLN_), or in combination with galloflavin (MSC_OLN+GF_) or transfected them with a siRNA against LDHA (MSC_OLN+siLDHA_) to decrease their lactate production (Fig. 4A). Our results showed that lactate efflux was increased by 275% in MSC_OLN_ as compared to MSC_CTL_ (Fig. 4A, B). This effect was partially reversed when the cells were treated with LDHA inhibitor (Fig. 4B, striped bar). Inducing freshly isolated CD4^+^ T cells to differentiate into Th1 or Th17 lymphocytes alone, or in the presence of the different MSC groups showed that MSC_OLN_ elicits a significantly higher immunosuppressive activity on Th1 and Th17 cells than MSC_CTL_ (Fig. 4C–F, striped, gray bar, and gray bar, respectively). However, the enhanced immunosuppressive effect exerted by MSC_OLN_ was significantly reduced when the cells were also treated with LDHA inhibitors (MSC_OLN+GF_ or MSC_OLN+siLDHA_) (Fig. 4C–F). These data showed that the stronger suppressive activity of glycolytic MSCs depends in part on lactate release.

### The immunosuppressive effect of human UC-MSCs depends on their lactate efflux

**Fig. 5:**
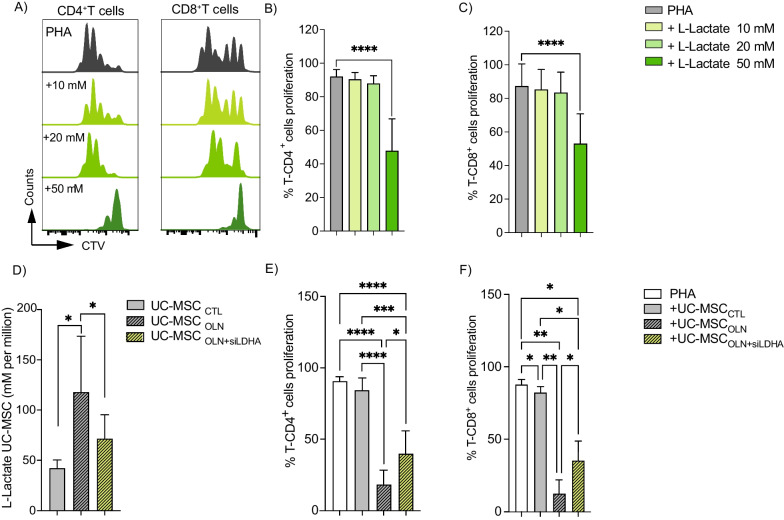
Lactate produced by UC-MSCs plays a critical role in their suppressive function. **A**–**C** PBMC were labeled with CTV and cultured alone or with 10, 20 or 50 mM L-lactate. After 4 days of culture, CD4^+^ (**B**) and CD8^+^ (**C**) T cells proliferation was analyzed by FACS. **D** Colorimetric analysis of lactate efflux by UC-MSCs after 24 h of treatment. **E**, **F** PBMCs labeled with CTV were cultivated alone (white bars) or co-culture with UC-MSC_CTL_ (gray bars), UC-MSC_OLN_ (striped, gray bars), or UC-MSC_OLN+siLDHA_ (striped, yellow bars). Proliferation of CD4^+^ (**E**) and CD8^+^ (**F**) T cells was assessed by flow cytometry. Results represent the mean ± SD of three independent experiments for lactate measurement in UC-MSCs and two independent experiments with at least six different biological samples for PBMCs; *p < 0.05, **p < 0.01, ***p < 0.001, ****p < 0.0001 (unpaired Kruskal–Wallis test). Unless otherwise indicated, comparisons were with UC-MSCs in basal or control conditions or activated PBMCs

We next assessed the effect of lactate produced by human MSCs on human T cells by first isolating PBMCs and labeling them with CTV prior to their culture with different concentrations of L-Lactate in the presence of PHA to activate T-Cells. FACS analysis revealed that L-Lactate did not significantly induce apoptosis at any of the L-Lactate concentrations analyzed (Additional file 6: Fig. S6A, B) although the proliferation of both CD4^+^ (Fig. 5A, B) and CD8^+^ (Fig. 5A, C) T cells was significantly reduced in the presence of 50 mM L-Lactate. Additionally, we compared the immunosuppressive effect of naïve UC-MSCs pretreated with OLN alone (UC-MSC_OLN_), to induce a glycolytic metabolism, with MSC knock-down for LDHA and pretreated with OLN (UC-MSC_OLN+siLDHA_) to inhibit the lactate production. UC-MSC_OLN_ had a significantly higher lactate efflux than naive UC-MSCs (UC-MSC_CTL_) (Fig. 5D, gray bar and striped, gray bar, respectively) that was correlated with a significantly increased immunosuppressive activity as compared to UC-MSC_CTL_ (gray bars) as shown by decreased proliferation of both CD4^+^ and CD8^+^ T cells (Fig. 5E, F, respectively). However, when LDHA was silenced in UC-MSC_OLN_, lactate efflux was significantly decreased (Fig. 5D, striped, yellow bar). These results were associated with a decrease in the enhanced suppressive activity in UC-MSC_OLN_ (striped, gray bars) when LDHA expression was inhibited. Indeed, the proliferation rate of both CD4^+^ and CD8^+^ T cells was greater (Fig. 5E, F, respectively; striped, yellow bar), resulting in a significant decrease in immunosuppressive activity. Altogether these data corroborate the critical role of lactate release in the suppressive activity of human- and mouse-derived glycolytic MSCs.

## Discussion

In the present work, we evaluated the role of lactate as a new mediator of immunoregulatory activities of both human and mouse MSCs, identifying a novel non-classical immunosuppressive mechanism. Lactate production by human and mouse MSCs was critical for their immunomodulatory functions in vitro, on CD4^+^ T cells, and in vivo, in a murine model of DTH. First, we observed that both Th1 and Th17 cells express MCT1 and MCT4 (although to a greater extent MCT4), two critical MCT that function as proton symporters that are stereoselective for L-Lactate. MCT1 is the most ubiquitously expressed family member with high affinity for lactate (*K*_m_, 7.7 mM) [11, 35]. Conversely, MCT4, a hypoxia-inducible MCT, has a high capacity for lactate transport (*K*_m_, 34 mM) and is expressed by cells with a high glycolytic metabolism [11, 35]. In addition, lactate serves as a signaling molecule through a G protein-coupled receptor, known as HCAR1 [36], which has been described as a receptor sensitive to L- and D-Lactate stereoisomers [37]. Interestingly, our immunofluorescence studies showed that HCAR1 is also expressed by Th1 and Th17 cells. Consequently, lactate can function as both a paracrine and autocrine signaling molecule, exerting its action on CD4^+^ T cells either in a receptor-dependent manner with several biological consequences or as an extracellular shuttle through MCT1 or MCT4. In this aspect, further studies will be necessary to evaluate the functional role of MCTs and lactate receptors in T cells.

We also observed that high external concentration of L-Lactate (25–50 mM) can dramatically affect Th1 and Th17 cell proliferation and differentiation. This immunosuppressive activity of lactate has been previously reported in a cancer context where tumor-derived lactic acid inhibits the proliferation of human T lymphocytes [38], decreases NK cell activity and induces M2 macrophage polarization [39, 40], suggesting that the production of lactic acid or L-Lactate by tumor cells may facilitate escape from immune surveillance [41]. Although the molecular pathways underlying lactate-related immunomodulation in autoimmunity and inflammation are still under investigation, there is some evidence that extracellular lactate levels directly affect T cell metabolism and cytokine production [42]. Indeed, Angelin et al. [31] demonstrated that 20 mM L-Lactate suppresses the proliferation of murine effector T cells, independent of acidity and toxicity [31]. Moreover, lactate alone can induce the plasticity of Th17 cells into Treg cells by functioning as a substrate of posttranslational modifications, leading to epigenetic changes in a process called histone lactylation [43, 44]. Through this process, lactate can also directly regulate the transcription of inflammatory genes and modulate the stability of anti-inflammatory transcription factors, such as FOXP3 [43–45]. In line with these results, we have recently demonstrated that the improved immunosuppressive activity of glycolytic MSCs was associated with the secretion of higher lactate levels compared to control MSCs, suggesting that this metabolite could be a potential mediator of the enhanced MSC immunosuppressive activity [7]. We have previously shown that activation of both murine BM-MSCs and human UC-MSCs with proinflammatory cytokines induced glycolysis and lactate production, and murine BM-MSCs release higher lactate levels in comparison to human UC-MSCs [7]. Variations in lactate levels produced could be due to differential expression of key glycolysis molecules, such as GLUTs, hexokinase isoforms, the NAD (+)/NADH redox state [46], as well as differences between tissue-specific MSCs or variations in different donor MSCs [47]. Remarkably, our in vitro and in vivo results showed that pharmacological and genetic inhibition of lactate secretion considerably impaired the immunosuppressive and therapeutic potential of MSCs by affecting Th1 and Th17 cell proliferation and differentiation potential and restraining inflammation in a murine model of DTH, demonstrating the role of lactate on MSC immunoregulatory function. Furthermore, pretreatment of MSCs with OLN together with lactate inhibitors (pharmacological and genetic) also impaired the enhanced immunosuppressive capacity displayed by glycolytic MSCs, validating the critical role of lactate on MSC suppressive function. Similarly, Selleri et al. [25], had previously reported that UC-MSCs abrogates monocyte differentiation into DCs by inducing cells with M2-macrophage features in terms of morphology, surface markers, migratory properties, and antigen presentation capacity [25]. This was partly mediated by lactate produced by UC-MSCs since the pharmacological inhibition of lactate on UC-MSCs partially reverses this effect [25]. Here, we showed that the genetic inhibition of LDHA in glycolytic UC-MSCs mitigated their immunotherapeutic potential in vitro on CD4^+^ and CD8^+^ human T cells, confirming the critical role of lactate release by human MSCs in their immunosuppressive function.

## Conclusion

**Fig. 6:**
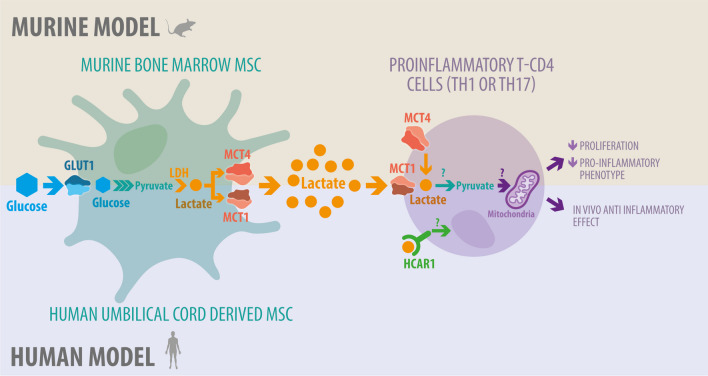
Graphical abstract. MSCs can exert their suppressive activity on human and murine T cells through an alternative lactate-dependent mechanism that is independent on classical suppressive factors. This suppressive activity improved when MSC are under a glycolytic metabolism due to an increase in lactate release leading to a restrain on in vivo inflammation

The results exhibited here propose for the first time a new MSC immunosuppressive pathway that is independent on the classical suppressive mechanism that depends on MSC-derived metabolites from glycolysis, such as lactate (Fig. 6).

### Supplementary Information

**Additional file 1: Fig. S1.** L-Lactate did not induce apoptosis of CD4^+^ T cells. Representative flow cytometry plots using Annexin V/PI staining for apoptosis (**A**). CD4^+^ T cells were treated for 3 days with different L-Lactate concentrations (10 mM, 20 mM and 50 mM) and then stained with Annexin V/PI for flow cytometric analysis. Percentage of live CD4^+^ T cells after L-lactate incubation (**B**). Percentage of apoptotic cells after treatment with different L-Lactate concentrations (**C**). After 3 days of culture with L-Lactate, Treg induction from Th1 (**D**) and Th17 (**E**) cells was analyzed by FACS. Results represent the mean ± SD of two independent experiments and four biological samples for CD4^+^ T cells; *p < 0.05 (unpaired Kruskal–Wallis test). Unless otherwise indicated, comparisons were with control conditions, or with Th1 or Th17 cells.

**Additional file 2: Fig. S2.** LDH knock-down and lactate inhibition with galloflavin does not change murine MSC metabolic activities. LDH knock-down in murine MSCs by a siRNA against LDH (MSC_siLDHA_) for 24 h was evaluated by qRT-PCR (**A**). The effect of the siRNA and galloflavin treatment in MSCs on the protein expression level of LDHA after 24 h was evaluated by Western blot (**B**). The metabolic profile of control MSCs (black) or incubated with galloflavin (GF, orange) or siRNA against LDH (siLDH, yellow) for 24 h was evaluated by measuring the oxygen consumption rate (OCR) (**B**) and the extracellular acidification rate (ECAR) (**C**).

**Additional file 3: Fig. S3.** Lactate inhibition does not affect the production of classical suppressive factors. The expression of classical suppressive factors VCAM, ICAM, and PD-L1 was evaluated in murine MSCs pretreated or not with galloflavin or an siRNA against LDH by FACS under basal conditions (**A**–**F**) or stimulated with the proinflammatory cytokines, TNFα and IFNγ (**G**–**L**). Nitric oxide (NO_2_) quantification on MSCs pretreated or not with galloflavin or a siRNA against LDH under basal or proinflammatory cytokines conditions (**J**). Results represent the mean ± SD of three independent experiments for MSCs. *p < 0.05, **p < 0.01, ***p < 0.001 (unpaired Kruskal–Wallis test). Unless otherwise indicated, comparisons were with MSCs in basal or control conditions.

**Additional file 4: Fig. S4.** Inhibition of lactate transporter MCT impairs the suppressive activity of murine MSCs in vitro on proinflammatory Th1 and Th17 cells. Naive CD4^+^ T cells from C57BL/6 mice were labeled with CTV and stimulated to differentiate to Th1 (**A**, **B**) or Th17 (**C**, **D**) cells and were cultured alone (Dark and light red, respectively) or with control MSCs (gray bar) or pretreated to inhibit lactate transporters with AZD3965 (green bar) or 4-CIN (light blue bar). After 3 days of co-culture, proliferation of Th1 (**A**) and Th17 (**C**) cells, and IFNγ (**B**) or IL-17 (**D**) production were measured by flow cytometry. Results represent the mean ± SD of two independent experiments and four biological samples for CD4^+^ T cells; *p < 0.05, **p < 0.01, ***p < 0.001 (unpaired Kruskal–Wallis test). Unless otherwise indicated, comparisons were with MSC in basal or control conditions, or with Th1 or Th17 cells.

**Additional file 5: Fig. S5.** Inhibition of lactate production in MSCs reduces their peripheral anti-inflammatory activity in a murine DTH model. After euthanasia, proinflammatory Th1 and Th17 lymphocytes (**A**, **B**), anti-inflammatory Treg cells (**C**) and Treg/Th17 ratio (**D**) were analyzed in the blood of DTH mice (black line bars) or DTH mice treated with MSC_CTL_ (gray line bars), MSC_GF_ (orange bars), or MSC_siLDHA_ (yellow bars) by FACS. Each color represents an independent DTH experiment (DTH1: in dark blue; DTH2: in red; DTH3 in yellow). Results represent the mean ± SD of at least five animals per experimental group; *p < 0.05, **p < 0.01, ***p < 0.001, ****p < 0.0001 (Unpaired ANOVA test). Unless otherwise indicated, comparisons were with untreated DTH mice.

**Additional file 6: Fig. S6.** L-Lactate did not induce apoptosis of human PBMCs. PBMCs were labeled with CTV, treated with different concentrations of L-Lactate (10 mM, 20 mM and 50 mM) in the presence of PHA to activate T cells, and then stained with Annexin V/PI for flow cytometric analysis. Percentage of live PBMCs after L-lactate incubation (**A**) and percentage of apoptotic cells after treatments with different L-Lactate concentrations (**B**). Results represent the mean ± SD of two independent experiments and four biological samples for PBMCs; *p < 0.05, **p < 0.01, ***p < 0.001 (unpaired Kruskal–Wallis test).

## Data Availability

The data that support the findings of this study are available in the methods of this article. Further information regarding the experimental design or the results obtained in this article are available on request from the corresponding author.

## Abbreviations

MSC: Mesenchymal stem/stromal cells
UC: Umbilical cord
LDH: Lactate dehydrogenase
CD: Cluster of differentiation
DTH: Delayed type 1 hypersensitivity
MCT: Monocarboxylate transporter
