# Supplementary figures and images for: Lactate: an alternative pathway for the immunosuppressive properties of mesenchymal stem/stromal cells
Source: Stem Cell Res Ther. 2023 Nov 19;14:335. doi: 10.1186/s13287-023-03549-4 (PMC10659074; doi:10.1186/s13287-023-03549-4)

Supplementary Figure 1

A)

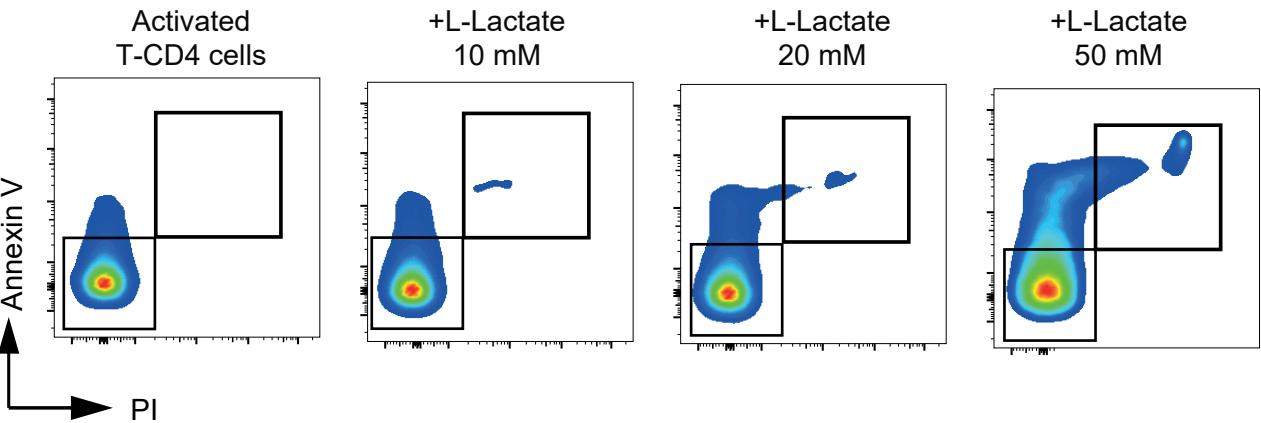

B)

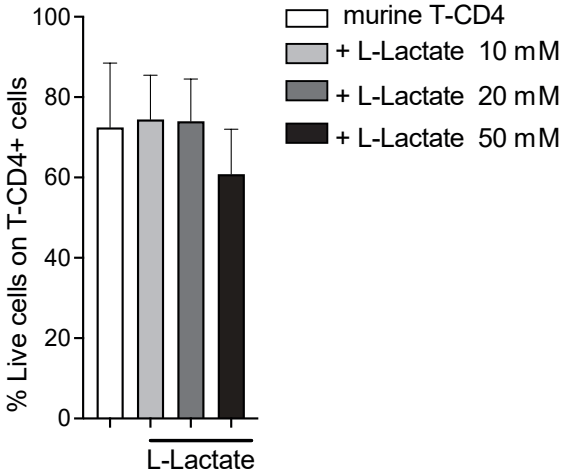

C)

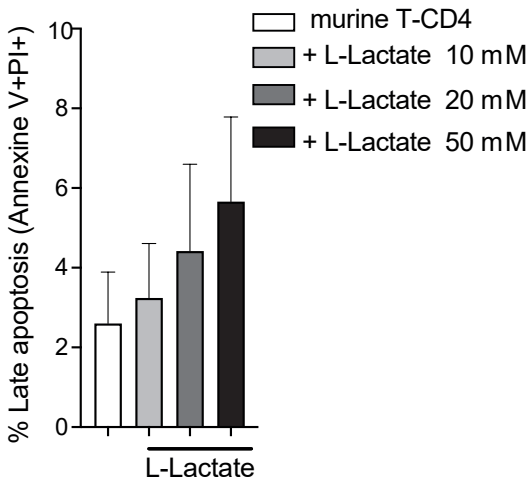

D)

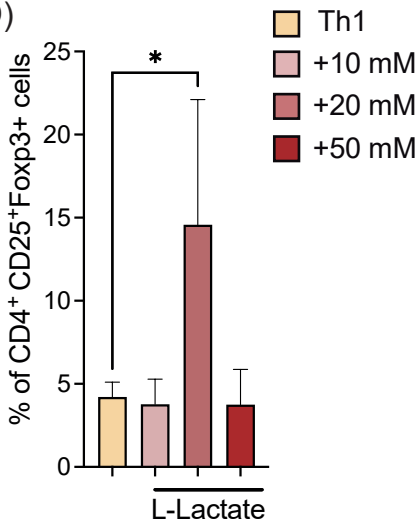

E)

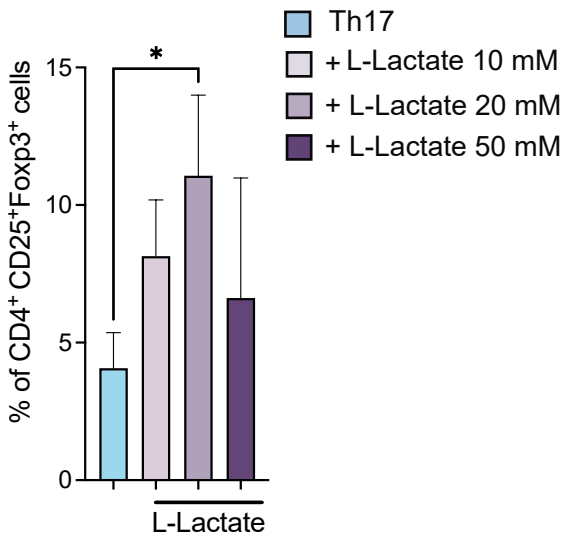

Supplement: Supplementary file 1 — Additional file 1: Fig. S1. L-Lactate did not induce apoptosis of CD4+ T cells. Representative flow cytometry plots using Annexin V/PI staining for apoptosis (A). CD4+ T cells were treated for 3 days with different L-Lactate concentrations (10 mM, 20 mM and 50 mM) and then stained with Annexin V/PI for flow cytometric analysis. Percentage of live CD4+ T cells after L-lactate incubation (B). Percentage of apoptotic cells after treatment with different L-Lactate concentrations (C). After 3 days of culture with L-Lactate, Treg induction from Th1 (D) and Th17 (E) cells was analyzed by FACS. Results represent the mean ± SD of two independent experiments and four biological samples for CD4+ T cells; *p < 0.05 (unpaired Kruskal–Wallis test). Unless otherwise indicated, comparisons were with control conditions, or with Th1 or Th17 cells. [file 13287_2023_3549_MOESM1_ESM.pdf]

Supplementary Figure 2

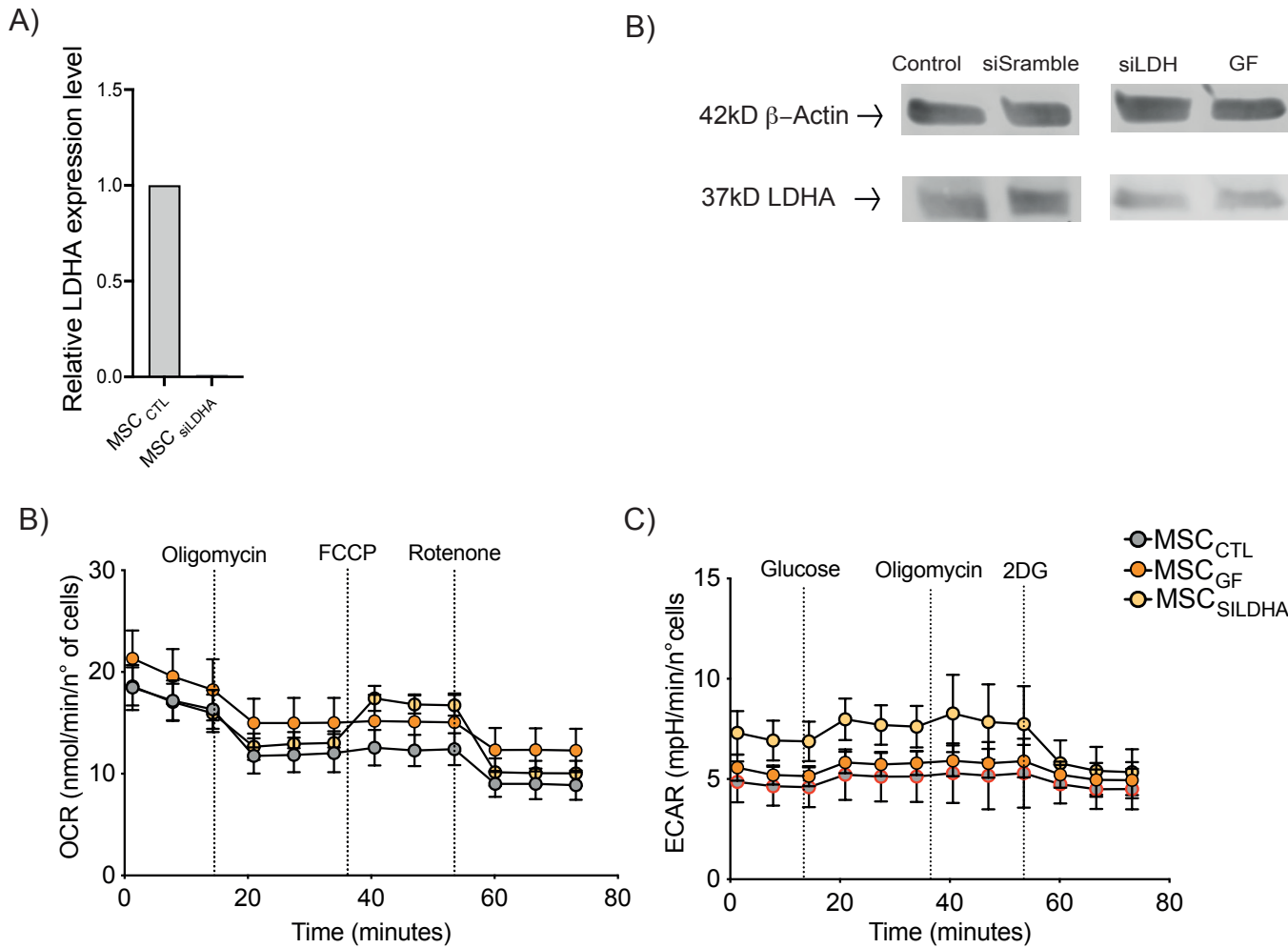

Supplement: Supplementary file 2 — Additional file 2: Fig. S2. LDH knock-down and lactate inhibition with galloflavin does not change murine MSC metabolic activities. LDH knock-down in murine MSCs by a siRNA against LDH (MSCsiLDHA) for 24 h was evaluated by qRT-PCR (A). The effect of the siRNA and galloflavin treatment in MSCs on the protein expression level of LDHA after 24 h was evaluated by Western blot (B). The metabolic profile of control MSCs (black) or incubated with galloflavin (GF, orange) or siRNA against LDH (siLDH, yellow) for 24 h was evaluated by measuring the oxygen consumption rate (OCR) (B) and the extracellular acidification rate (ECAR) (C). [file 13287_2023_3549_MOESM2_ESM.pdf]

Supplementary Figure 3

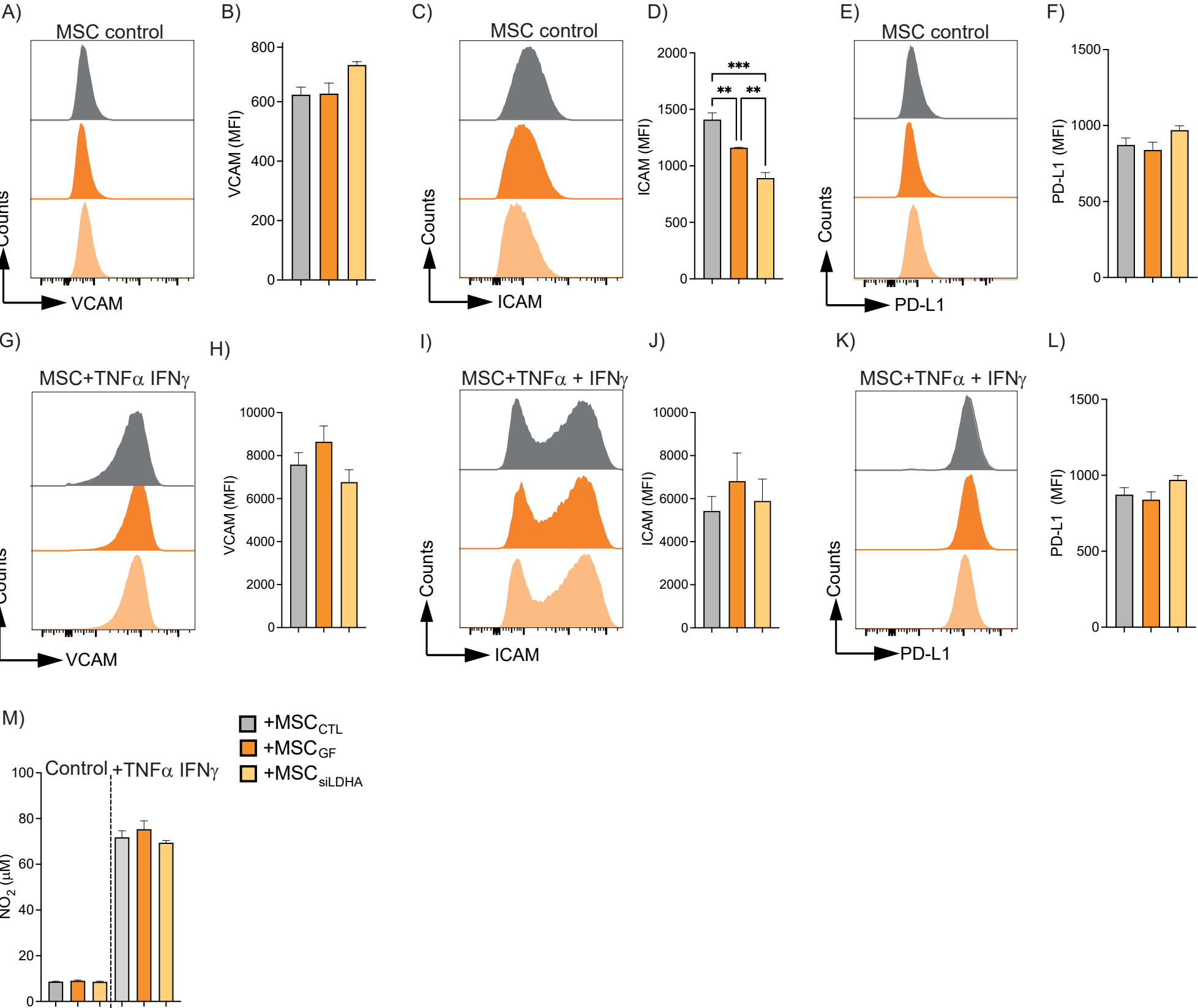

Supplement: Supplementary file 3 — Additional file 3: Fig. S3. Lactate inhibition does not affect the production of classical suppressive factors. The expression of classical suppressive factors VCAM, ICAM, and PD-L1 was evaluated in murine MSCs pretreated or not with galloflavin or an siRNA against LDH by FACS under basal conditions (A–F) or stimulated with the proinflammatory cytokines, TNFα and IFNγ (G–L). Nitric oxide (NO2) quantification on MSCs pretreated or not with galloflavin or a siRNA against LDH under basal or proinflammatory cytokines conditions (J). Results represent the mean ± SD of three independent experiments for MSCs. *p < 0.05, **p < 0.01, ***p < 0.001 (unpaired Kruskal–Wallis test). Unless otherwise indicated, comparisons were with MSCs in basal or control conditions. [file 13287_2023_3549_MOESM3_ESM.pdf]

Supplementary Figure 4

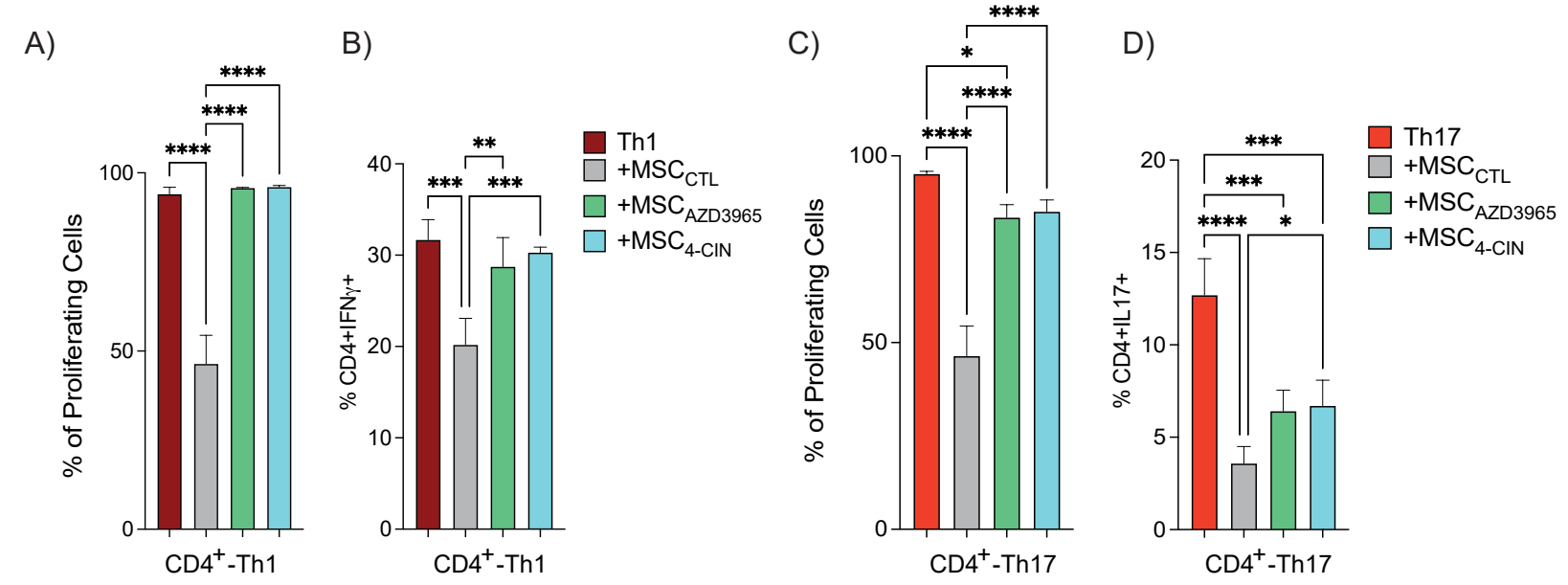

Supplement: Supplementary file 4 — Additional file 4: Fig. S4. Inhibition of lactate transporter MCT impairs the suppressive activity of murine MSCs in vitro on proinflammatory Th1 and Th17 cells. Naive CD4+ T cells from C57BL/6 mice were labeled with CTV and stimulated to differentiate to Th1 (A, B) or Th17 (C, D) cells and were cultured alone (Dark and light red, respectively) or with control MSCs (gray bar) or pretreated to inhibit lactate transporters with AZD3965 (green bar) or 4-CIN (light blue bar). After 3 days of co-culture, proliferation of Th1 (A) and Th17 (C) cells, and IFNγ (B) or IL-17 (D) production were measured by flow cytometry. Results represent the mean ± SD of two independent experiments and four biological samples for CD4+ T cells; *p < 0.05, **p < 0.01, ***p < 0.001 (unpaired Kruskal–Wallis test). Unless otherwise indicated, comparisons were with MSC in basal or control conditions, or with Th1 or Th17 cells. [file 13287_2023_3549_MOESM4_ESM.pdf]

Supplementary Figure 5

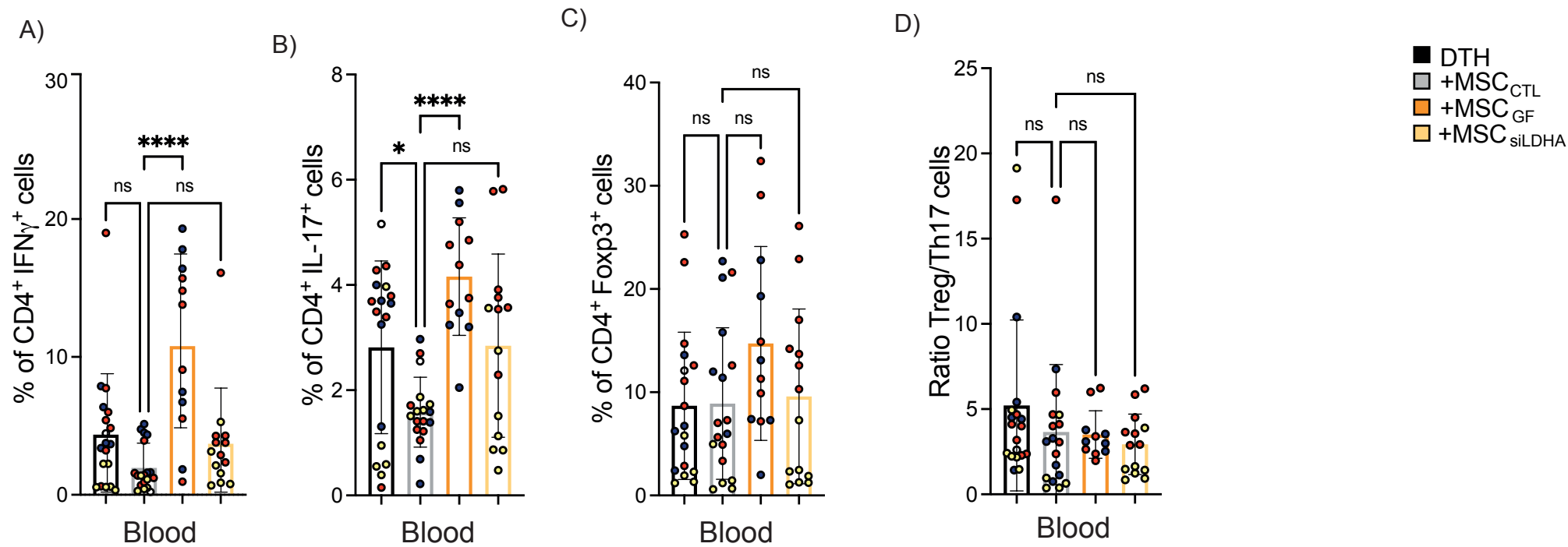

Supplement: Supplementary file 5 — Additional file 5: Fig. S5. Inhibition of lactate production in MSCs reduces their peripheral anti-inflammatory activity in a murine DTH model. After euthanasia, proinflammatory Th1 and Th17 lymphocytes (A, B), anti-inflammatory Treg cells (C) and Treg/Th17 ratio (D) were analyzed in the blood of DTH mice (black line bars) or DTH mice treated with MSCCTL (gray line bars), MSCGF (orange bars), or MSCsiLDHA (yellow bars) by FACS. Each color represents an independent DTH experiment (DTH1: in dark blue; DTH2: in red; DTH3 in yellow). Results represent the mean ± SD of at least five animals per experimental group; *p < 0.05, **p < 0.01, ***p < 0.001, ****p < 0.0001 (Unpaired ANOVA test). Unless otherwise indicated, comparisons were with untreated DTH mice. [file 13287_2023_3549_MOESM5_ESM.pdf]

Supplementary Figure 6

A)

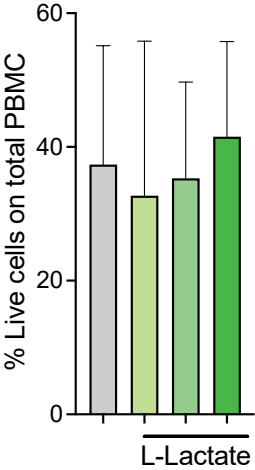

B)

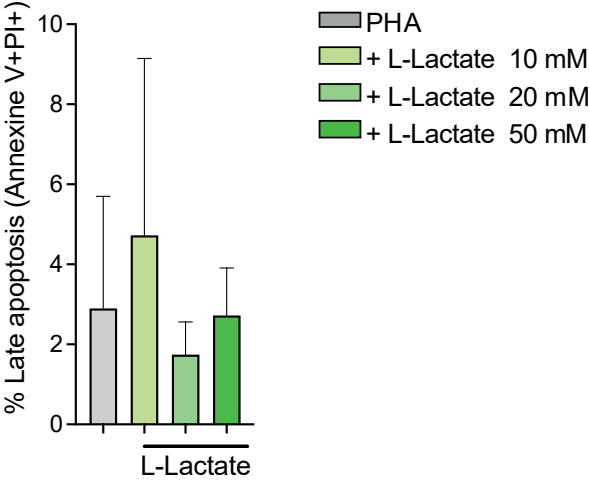

Supplement: Supplementary file 6 — Additional file 6: Fig. S6. L-Lactate did not induce apoptosis of human PBMCs. PBMCs were labeled with CTV, treated with different concentrations of L-Lactate (10 mM, 20 mM and 50 mM) in the presence of PHA to activate T cells, and then stained with Annexin V/PI for flow cytometric analysis. Percentage of live PBMCs after L-lactate incubation (A) and percentage of apoptotic cells after treatments with different L-Lactate concentrations (B). Results represent the mean ± SD of two independent experiments and four biological samples for PBMCs; *p < 0.05, **p < 0.01, ***p < 0.001 (unpaired Kruskal–Wallis test). [file 13287_2023_3549_MOESM6_ESM.pdf]
